# The Influence of Perception of Social Equality and Social Trust on Subjective Well-Being Among Rural Chinese People: The Moderator Role of Education

**DOI:** 10.3389/fpsyg.2021.731982

**Published:** 2022-01-03

**Authors:** Shuang Gao, Jilun Zhao

**Affiliations:** School of Political Science and Law, Northeast Normal University, Changchun, China

**Keywords:** perception of social equality, social trust, subjective well-being, rural China, education

## Abstract

The present study explored the moderation effect of education on the relationship between the perception of social equality and social trust and individuals’ subjective well-being (SWB) in rural China. Data were derived from the nationally representative cross-sectional Chinese General Social Survey (CGSS). After handling missingness, 5,911 eligible participants (age 18 years or older) from the 2015 wave were included in the model. We used logistic regression to test the hypotheses. We first tested the effect of the perception of social equality and social trust on SWB. Then we added an interaction term to test the moderation effect of education in this relationship. The results show that education had a significant moderating effect on the association between general social trust and SWB. While had no significant effect on the association between the perception of social equality, special social trust and SWB. The perception of social equality had significant effects in both groups. The relationship between special social trust and SWB in both groups was not significant. For the more educated group, general social trust had a significant and positive effect on SWB.

## Introduction

In recent years, subjective well-being (SWB) has gained much attention. SWB contributes to lifelong health (Ortega et al., 2010; Boehm et al., 2011). As a public health goal, SWB defined as the cognitive judgment and affective evaluation of one’s life (Cruwys et al., 2013; Forsman et al., 2013). The evaluations included emotional reactions to events and the cognitive judgments to satisfaction and fulfillment (Diener et al., 2002). From this perspective, SWB is a multidimensional concept that contains the experiences of pleasant emotion, lower level of negative moods and higher life satisfaction (Diener et al., 2002). The importance of SWB is well recognized by researchers and policy makers, the attributing elements of SWB in existing studies included optimal health, health relationships and quality of life (Helliwell and Wang, 2011; Klein, 2013; Churchill and Mishra, 2017). In addition, SWB has been frequently examined by extensive research, with evidence indicating that some demographic factors, such as age, gender, educational attainment, socioeconomic status, income, and marriage status significantly affect SWB; furthermore, an individual’s SWB is also strongly shaped by their cognition to the surrounding life environment, such as their social trust in others and perception of social equality (Young et al., 2004; Agampodi et al., 2015; Flores et al., 2018; Xiao et al., 2020).

The perception of social equality is a type of subjective psychological feeling regarding how people judge social equality issues; it is an evaluation related to the distribution of important social resources (Yu and Zhang, 2018). The evaluation usually is generated by comparing individuals’ reputation, socioeconomic status, and financial status with others and is closely related to individuals’ living environments, cultural background, and value proposition (Christopher, 2004). The perception of social equality could reflect an individual’s assessment of their current living environment and further influence their health and SWB (Hu and Shen, 2015). The perception of social equality is an indicator of health and has a significant impact on individuals’ SWB (Hu and Shen, 2015).

As a developing country, China’s political and social institutions in various domains are under development. This situation causes uneven distribution of benefits and influences perceptions of social equality (Li, 2020). For instance, in rural China, affected by the binary societal system, the social institutional systems in rural areas are more defective than urban areas (Wang, 2011; Zhang et al., 2013; Yang and Xue, 2016; Zheng, 2018). People live in urban areas could enjoy comprehensive social security, including but not limited endowment insurance, medical insurance, maternity insurance, employment injury insurance, unemployment insurance and other social assistance and social welfare programs (Zhang et al., 2013; Zheng, 2018). While those who live in rural areas could only enjoy the most basic social security which meets the minimum standards required by the state, the endowment insurance, medical insurance, and other social security programs of people living in rural areas are far less than their counterparts in urban areas (Chen and Wang, 2011; Zhang et al., 2013). In addition, in rural areas, the living environments are relatively tougher, the economic development is lagging, and people have lower income and savings, those are highly related to individuals’ social equality perception (Ma and Liu, 2010; Yu and Zhang, 2018). In this situation, people in rural areas are more likely to perceive inequality (Huhe et al., 2015). According the discussion above, we proposed the first hypothesis:

H1. Perception of social equality significantly affects SWB in rural China.

Social trust is a kind of belief that affords honesty, integrity, and reliability to others, at the same time, views others as sharing the same interests as you and not doing you harm (Newton, 2013; Joseph and Shirley, 2015). Broadly defined in the literature, social trust is a dimension of social capital, some researches focus on social trust as the most component of social capital and test its effect on SWB, the results show that as an element of social capital, social trust has positive effect on SWB (Helliwell and Wang, 2011; Ram, 2010; Klein, 2013; Zhang et al., 2021). Social trust is individuals’ subject cognation to firsthand experiences with people in social world: family members, friends, relatives, neighbors, colleagues, and other daily contacts, is an important factor in social communication, deemed a crucial synthetic force of society, and its importance to human beings and society cannot be overemphasized (Joseph and Shirley, 2015). These contacts can be divided into two forms: (a) family members, relatives and familiar friends, defined as special social trust; and (b) social organizations, defined as general social trust (Francis, 1996; Porta et al., 1997; Nannestad, 2008; Delhey et al., 2011). Special social trust refers to individuals’ positive expectations for others in their own group or with whom they interact intimately (Michael et al., 2005). While general social trust refers to individuals’ positive expectations for ones they are not familiar with, outside their social group or with whom they do not interact directly (Toshio et al., 1998; Uslaner and Conley, 2003). Weber (1951) proposed that the trust of Chinese people is built on kinship or purely personal relationship, for those who are outside their kinship or personal relationship, they tend to distrust them (Weber, 1951). And Francis (1996) further quoted and extended this assertion. While some Chinese researchers tested the viewpoints of Weber (1951) and Francis (1996) pragmatically, and found that in the special context of China, special social trust and general social trust are not a kind of binary opposition thinking (Zhai, 2014). At the same time, with the rapid changes of China’s social structure, the life styles, communication styles and social values of Chinese people have changed greatly, although there is still an emphasis on friendship and family in China, while the essence works for Chinese people’s trust is not the relationship itself, but the psychological and emotional intimacy between the two sides contained in the relationship (Li and Liang, 2002; Liu, 2016; Churchill and Mishra, 2017). Thus, for Chinese people’s trust, there is not only a particular trust based on the personal relationship, and also a general trust based on common ideas or beliefs which is characterized by helping to promote social trust in a broader sense and improving integration throughout society (Li and Liang, 2002; Wang and Wang, 2021). According to this situation, we proposed the second hypothesis:

H2. Both of special and general social trust significantly affect SWB in rural China.

Previous studies have concluded that education has a significant influence on social equality, social trust, and SWB (Jian et al., 2011; Newton, 2013; Ye and Liang, 2016; Zhang et al., 2019). Several studies have recognized the importance of education for the development of SWB (Neugebauer et al., 2014). For instance, researchers posited strong relationships among social trust and personal demographic variables, such as education, income, social status, life satisfaction, happiness, insecurity, and anxiety (Putnam, 2000). Other studies have asserted that people with higher education perceive lower levels of social inequality and exhibit higher levels of social trust, because receiving more education may enhance individuals’ ability to interpret information, handle risk, and understand the actions of others (Huang et al., 2011; Jennifer et al., 2016; Luca, 2017; Sven et al., 2017; Yang, 2019). Furthermore, scholars have argued that education could help people “cultivate a more benign view of the world,” which is important for enhancing social trust and social equality (Huang et al., 2011; Luca, 2017). A research based on the data from 2005 to 2015 Chinese General Social Surveys concluded that, with the higher the education, the higher the SWB, and there was a significant positive relationship between education level and SWB (Jin et al., 2020). Evidences showed that people with higher education level may have better financial status and social status, they may have more resources to share with others and thus help them to extend their social networks, and further promote their SWB (Churchill and Mishra, 2017; Zhang et al., 2019). However, most studies related to social equality, social trust, and SWB have used education as a control variable or analyzed the influence of education level to SWB directly. This study examined the relationship of perception of social equality and social trust with SWB and explored the effect of education level in these associations. According to previous studies about the relationship among perception of social equality, social trust and SWB, the following hypotheses were proposed:

H3. Education has an interaction effect in the association between perception of social equality and SWB.

H4. Education has an interaction effect in the association between special and general social trust and SWB.

## Materials and Methods

### Sampling

The data of the study were derived from Chinese General Social Survey (CGSS). CGSS is a nationally representative, cross-sectional sample of individuals in mainland China that was launched in 2003 and is publicly available. The survey was conducted by the Department of Sociology of Renmin University of China, which systematically collected multilevel data, including social, community, family, and individual data, that aimed to summarize the tendency of social transformation. The baseline of CGSS was performed in 2003. Multistage stratified probability-proportionate-to-size sampling was used to survey about 10,000 individuals in 125 counties.

For this analysis, the 2015 wave of the survey was used, in which 10,968 respondents from 28 provinces had completed the questionnaire. The research focus in this analysis was individuals in rural China; thus, we selected respondents from agricultural households as the sample. After handling missingness, 5,911 eligible participants from the 2015 wave were included in the model.

### Measurements

#### Dependent Variable

In this analysis, SWB was the outcome variable. Respondents indicated how they viewed their SWB using a 5-point Likert scale (1 = very bad, 3 = average, and 5 = very good). A total of 59.3 and 16.4% of the respondents reported that their SWB was good and very good, respectively. In contrast, 9.0% rated their SWB levels as “bad/very bad,” and 15.3% rated “average.” In order to adjust for the skewed distribution of SWB, the responses were further recoded into a binary variable (0 = very bad/bad/average; 1 = good/very good). Such method was found to be valid measurement tool to test variables with skewed distributions (Lucas and Donnellan, 2012; Li et al., 2015; Lu et al., 2021).

#### Independent Variable

Social trust and perception of social equality were used as the independent variables. The respondents were asked about social trust as follow: “If it does not directly involve monetary interests, in general social interactions and contacts, to what extent will you trust people as follows: relatives, residents in the same village, people with the same or different family name, colleagues, old schoolmates, people who participate in entertainment, religious, or social activities together.” Responses were measured on a 5-point Likert scale (1 = majority are unreliable, 5 = majority are reliable). Different from western culture of individualism, China is a kind of relationship society based on situationism, and for Chinese people, the standards of classifying special trust and general trust cannot be copied from the western standards (Li and Liang, 2002). For Chinese people, their special social trust refers to the trust in ones they contact frequently, including consanguinity group, geographical group, and interest group so forth. And general social trust refers to the trust in ones they are unacquainted or not contact very frequently (Wang and Wang, 2021). According to the discussion above and the results of exploratory factor analysis and confirmatory factor analysis (the results of confirmatory factor analysis were presented in the Appendix), we further divided social trust as special social trust and general social trust (Ross et al., 2014). Special social trust included trust in relatives and people who engage in entertainment, religious, and social activities with the respondents. A sum score was calculated (Cronbach’s alpha = 0.767), the theoretical range of values are from 1 to 20. General social trust included residents in the same village, people with the same or different family name as the respondents, colleagues, and old schoolmates. A sum score was calculated (Cronbach’s alpha = 0.844), the theoretical range of values are from 1 to 25.

Perception of social equality was also used as an independent variable. The respondents were asked, “Generally speaking, do you think current society is equal?” A 5-point Likert scale was used to measure answers, from 1 (completely unfair) to 5 (completely fair).

#### Moderator Variable

The moderating variable in this analysis was education; in the survey questionnaire, educational attainment ranged from illiterate to post-graduate or above. To analyze the moderator role of education, we divided the respondents into two groups, recoding education as a dichotomous variable: 0 represents people with an education level of primary school or less, and 1 represents people with an education level of secondary school or more.

#### Control Variables

According to previous studies, the demographic factors could affect SWB significantly (Young et al., 2004; Agampodi et al., 2015; Flores et al., 2018; Xiao et al., 2020; Zhang et al., 2021). Thus, we added age, gender, marital status, self-rated economic status, political status, ethnicity, and self-rated social class status into the model as control variables. Age was calculated as the respondents’ birth year subtracted from 2015. Gender was a dichotomous variable (0 = male, 1 = female). Marital status, ethnicity, and political status were recoded as dichotomous variables (marital status: 0 = other, 1 = married; ethnicity: 0 = Han, 0 = other; and political status: 0 = other, 1 = communist or communist Youth League member). Self-rated class status was measured by asking participants to indicate their social class (1 = lowest level, 10 = highest level). Self-rated economic status was measured by asking “In contrast to your peers, how do you view your economic status?” Three answer options were available (1 = far lower than average, 3 = average, and 5 = far higher than average), we further recoded the variable as a binary variable (0 = lower than average, 1 = equal or higher than average).

#### Data Analysis

Logistic regression was applied to test the hypotheses using SPSS20.0. Education was used as a moderator. First, we established an overall model measuring the influence of perception of social equality and social trust on SWB. Second, we explored the interaction effect of education in the relationship of perception of social equality and social trust with SWB. In the final step, we examined the effects of perception of social equality and social trust on SWB in lower and higher education groups separately. Nagelkerke’s *R*^2^ and Hosmer and Lemeshow tests were used to test the model fit.

## Results

### Descriptive Statistics

Participants’ characteristics are summarized in Table 1. The average age was 50.3 years. In the sample, men accounted for 46% and women accounted for 54%. More than 80% of the respondents were married. Around 57.7% of the respondents considered their economic status to exceed the average level. More than 90% of the respondents did not participate in the Communist Party of China (member of the Communist Youth League). Participants with an education level of primary school or less accounted for 52.5% of the respondents; the rest had a secondary school education or more. An overwhelming majority of the respondents (close to 90%) were Han ethnicity. Around 75% of the respondents reported that their SWB was higher than average. The mean scores of self-rated of social class status and perception of social equality were 4.1 and 3.2, respectively. The average scores of general trust and special trust were 16.6 and 10.2, respectively.

**TABLE 1:** Sample characteristics (*N* = 5,911).

	*N* (%)	Mean (SD)	Value range
Age		50.3 (16.4)	18∼94
**Gender**			
Male	2,719 (46.0)		
Female	3,192 (54.0)		
**Marital status**			
Married	4,783 (80.9)		
Other marital status	1,128 (19.1)		
**Self-rated economic status**			
Lower than average	2,483 (42.0)		
Equal or higher than average	3,409 (57.7)		
**Political status**			
Communist (Youth League) member	490 (8.3)		
Other status	5,395 (91.3)		
**Educational level**			
Primary school education and lower	3,104 (52.5)		
Secondary school education and above	2,802 (47.4)		
**Ethnicity**			
Han	5,315 (89.9)		
Other	581 (9.8)		
**SWB**			
Lower	1,436 (24.3)		
Higher	4,473 (75.7)		
Self-rated social class status		4.1 (1.6)	1∼10
The perception of social equality		3.2 (1.0)	1∼5
Special social trust		10.2 (4.3)	1∼20
General social trust		16.6 (4.2)	1∼25

In this part, we also tested the correlations among independent variables and dependent variable. For SWB and binary independent variables, we used Chi-square tests to examine their correlations (Table 2). The results showed that the correlations between marital status, self-rated economic status, political status, educational level, and SWB were significant.

**TABLE 2:** Chi-square tests among subjective well-being (SWB) and binary independent variables.

	SWB				Chi-square	Sig
	Lower	%	Higher	%		
**Gender**					1.131	0.288
Male	678	24.9	2,040	75.1		
Female	758	23.8	2,433	76.2		
**Marital status**					26.849***	0.000
Married	1,095	22.9	3,687	77.1		
Other marital status	341	30.3	786	69.7		
**Self-rated economic status**					420.441***	0.000
Lower than average	936	37.7	1,545	62.3		
Equal or higher than average	495	15.5	2,914	85.5		
**Political status**					19.334***	0.000
Communist (Youth League) member	79	16.2	410	83.8		
Other status	1,352	25.1	4,042	74.9		
**Educational level**					41.407***	0.000
Primary school education and lower	859	27.7	2,244	72.3		
Secondary school education and above	574	20.5	2,227	79.5		
**Ethnicity**					1.289	0.256
Han	1,277	24.0	4,036	76.0		
Other	152	26.2	429	73.8		

***p < 0.001 (two-tailed).

For SWB and continuous independent variables, we used Pearson correlation tests to examine their correlations (Table 3). The results showed that the correlations between self-rated social class status, the perception of social equality, special social trust, general social trust, and SWB were significant.

**TABLE 3:** Correlations among subjective well-being (SWB) and continuous independent variables.

		SWB	Age	Class	Social equality	Special ST	General ST
SWB	Pearson correlation	1.000					
	Sig						
Age	Pearson correlation	–0.014	1.000				
	Sig	0.274					
Class	Pearson correlation	0.251***	−0.038*	1.000			
	Sig	0.000	0.004				
Social equality	Pearson correlation	0.252***	0.160***	0.148***	1.000		
	Sig	0.000	0.000	0.000			
Special ST	Pearson correlation	0.066***	−0.059***	0.052***	0.053***	1.000	
	Sig	0.000	0.000	0.000	0.000		
General ST	Pearson correlation	0.110***	−0.108***	0.067***	0.132***	0.432***	1.000
	Sig	0.000	0.000	0.000	0.000	0.000	

*p < 0.05 (two-tailed), ***p < 0.001 (two-tailed). Class = Self-rated social class status; Social equality = The perception of social equality; Special ST = Special social trust; General ST = General social trust.

### Results of Logistic Regression

In this part, we built two models. The results were presented in Table 4. In the first model, we added the perception of social equality and social trust into the model as independent variables, while also adding control variables. The value of Nagelkerke *R*^2^ was 21.6%, the result of the Hosmer and Lemeshow test was not significant. The perception of social equality and general social trust had a significant impact on participants’ SWB (the perception of social equality: *p* < 0.001, OR = 1.686; general social trust: *p* < 0.01, OR = 1.024); however, special social trust had no significant impact on SWB. For fully testing the research hypotheses, we examined the effect of special social trust on SWB without adding general social trust in the model, the results show that special social trust had significant influence on SWB without adding general social trust (*p* < 0.05, OR = 1.020).

**TABLE 4:** Logistic regression for subjective well-being (SWB).

Variables	1			2		
	OR	CI (95%)	Sig	OR	CI (95%)	Sig
(Constant)	3.913		0.000	3.913		0.000
Age	1.004	0.999–1.008	0.145	1.004	0.999–1.008	0.155
Gender	1.155*	1.007–1.323	0.039	1.145	0.999–1.312	0.053
Marital status	1.497***	1.271–1.763	0.000	1.516***	1.287–1.787	0.000
Self-rated economic status	2.445***	2.124–2.815	0.000	2.451***	2.129–2.823	0.000
Political status	1.512**	1.139–2.008	0.004	1.489*	1.121–1.978	0.006
Educational level	1.396***	1.188–1.641	0.000	1.376***	1.165–1.625	0.000
Ethnicity	0.911	0.734–1.131	0.397	0.900	0.725–1.118	0.342
Self-rated class status	1.258***	1.203–1.135	0.000	1.255***	1.201–1.312	0.000
Perception of social equality	1.686***	1.578–1.801	0.000	1.679***	1.571–1.794	0.000
Special social trust	1.010	0.993–1.028	0.242	1.011	0.994–1.029	0.216
General social trust	1.024**	1.006–1.042	0.009	1.027**	1.009–1.045	0.003
Perception of social equality *higher educational level				0.904	0.792–1.031	0.131
Special social trust *higher educational level				0.996	0.961–1.031	0.816
General social trust *higher educational level				1.058**	1.021–1.096	0.002

*p < 0.05 (two-tailed), **p < 0.01 (two-tailed), ***p < 0.001 (two-tailed).

### Moderation Effect of Education

**FIGURE 1:**
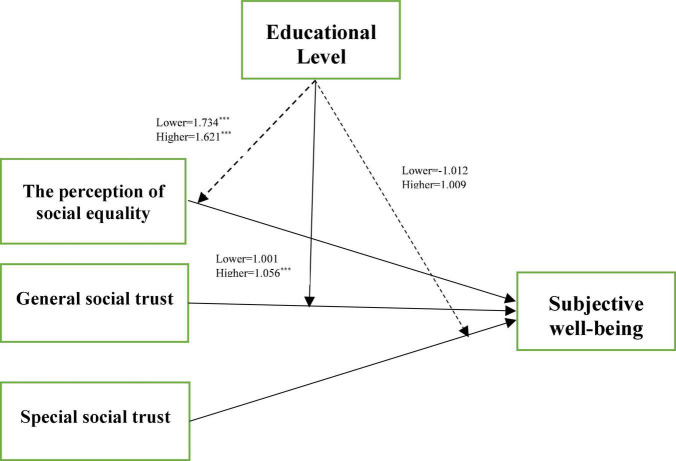
The moderator role of education in the association between perception of social equality, social trust, and SWB. Odds ratio values are reported. The full lines indicate moderating effect. The dashed line indicates no moderating effect. ^***^p < 0.001 (two-tailed).

In the second model, we tested the moderating role of education level. For avoiding the problem of multicollinearity in moderation model, we centered the predict variables. After centered the predictors, the coefficients of the variables did not change except the constants. This proved that the centralized model has not been mis-operated. The Nagelkerke *R*^2^ of the two models were 24.1 and 18.0%, respectively. The results of the Hosmer and Lemeshow test of the two models were not significant. The results showed that education level had a significant moderating effect in the relationship between general social trust and SWB (*p* < 0.01, OR = 1.058), but no significant moderating effect in the relationship between perception of social equality, special social trust and SWB (Figure 1).

We further divided the variable of education into two groups, lower (primary school or less) and higher (secondary school or more), to test the impact of perception of social equality and special and general social trust on participants’ SWB in both groups. The results were presented in Table 5. In the lower-education group, general social trust was not significantly associated with SWB, whereas in the higher-education group, it had a significant relationship (*p* < 0.001, OR = 1.056). Regarding the perception of social equality, it was significantly associated with SWB in both groups, and for special social trust, it had no significant effect on SWB in both groups.

**TABLE 5:** Moderation effects of education.

Variables	Lower educational level			Higher educational level		
	OR	CI (95%)	Sig	OR	CI (95%)	Sig
(Constant)	3.270		0.000	4.157		0.000
Age	1.009*	1.002–1.016	0.013	0.996	0.988–1.003	0.282
Gender	1.180	0.981–1.420	0.079	1.074	0.874–1.318	0.501
Marital status	1.519***	1.208–1.910	0.000	1.751***	1.342–2.284	0.000
Self-rated economic status	2.738***	2.258–3.320	0.000	2.121***	1.720–2.616	0.000
Political status	1.381	0.716–2.666	0.336	1.485*	1.079–2.043	0.015
Ethnicity	1.044	0.792–1.377	0.758	0.721	0.508–1.022	0.066
Self-rated class status	1.296***	1.220–1.377	0.000	1.204***	1.128–1.286	0.000
Perception of social equality	1.734***	1.584–1.898	0.000	1.621***	1.469–1.789	0.000
Special social trust	1.012	0.990–1.035	0.226	1.009	0.982–1.037	0.512
General social trust	1.001	0.978–1.025	0.919	1.056***	1.029–1.085	0.000

*p < 0.05 (two-tailed), ***p < 0.001 (two-tailed).

## Discussion

In this study, we focused on the perception of social equality, social trust, and SWB in rural China. We explored the significant impact of the perception of social equality and social trust on SWB and differences in their influences on SWB by education level. The results indicate that the perception of social equality and general social trust had significant effects on individuals’ SWB, which conformed to the theoretical hypotheses. While special social trust had no significant effect on SWB, this is not conformed to our theoretical hypotheses. For fully testing the theoretical hypotheses, we examined the effect of special social trust on SWB without control general social trust, the results showed that special social trust affects SWB significantly without added general social trust. While when we added general social trust into the model, special social trust became non-significant. In the context of Confucianism, Chinese people emphasis on family values strongly, for Chinese people, the trust to their families and relatives is naturally (Zhai, 2014). However, social trust needs to store abundant “stock” to establish general social trust to improve and integrate society, and special social trust could form the “stock” of general trust (Wang and Wang, 2021). The trusted people constitute the “trust circle” of the trust subject, as the circle expands, the objects of special social trust increase and reinforce the role of special social trust as “stock” (Wang and Wang, 2021). Thus, when the “stock” of special social trust is enough to become general social trust, general social trust will act instead of the role of special social trust to influence individuals’ health outcomes, including SWB.

Previous studies identified the positive effects of the perception of social equality and social trust on individuals’ SWB (Paul and Samantha, 2009; Zhao, 2012; Yuan and Golpelwar, 2013; Bian et al., 2018), and some studies proposed the important role of education (Toots and Lauri, 2015; Sven et al., 2017; Yang, 2019). The findings of present study provide additional evidence that the perception of social equality and general social trust play important roles in individuals’ SWB. Education level had no moderating effect in the association between the perception of social equality and SWB. The perception of social equality had significant influence in both education groups. In the whole model, the perception of social equality had significant impact on SWB, and in the moderation model, the perception of social equality had significant impact on SWB in both groups. This illustrated that the perception of social equality is important for all people, whatever their education level. Education level also had no moderator effect in the association between special social trust and SWB, we had discussed above, for Chinese people, special social trust is a kind of naturally status, it has not been activated, thus, people could even not perceive the exist of special social trust to influence their SWB (Zhai, 2014).

In addition, education level played a moderator role in the association between general social trust and SWB, which conformed to the theoretical hypotheses. In lower education level group, general social trust had no significant influence on SWB, while in the higher education level group, general social trust affected SWB significantly. People with more education may have better financial status, social status, and social network, further enhancing their ability to communicate with society and extending their “trust circle,” thus, general social trust could play important role to their SWB. Whereas for people with lower education level, their lower financial status and social status may limit their communication with others, and further influence their SWB (Churchill and Mishra, 2017; Zhang et al., 2019).

The findings emphasize the importance of pursuing future policies that develop more socially equal communities. At the same time, for people with lower education level, government and communities should focus on helping them to extend their social capital, promote their general social trust to enhance their SWB and further promote the sustainability and development of rural Chinese areas.

This study has some limitations. First, the CGSS data were cross-sectional; generally, causal relationships cannot be tested through analysis of cross-sectional data. However, the study was based on previous research and related theories, thus, the results of the study are convincing. Second, the survey did not control all potential covariates including respondents’ social participation, political attitudes and the like which may confound the results of the model, and it is difficult to eliminate all confounding effects. Third, economic status and social class status were both self-reported in the survey, which may lead to information accuracy.

## Conclusion

The present study explored the moderating role of education in the association between the perception of social equality and social trust and individuals’ SWB in rural China. The findings suggest that education has a moderating effect in the relationship between general social trust and SWB. The effect of the perception of social equality on SWB was significant in both education groups, whereas the effect of general social trust only existed in the higher-education group. Education had no moderating effect in the relationship between special social trust and SWB.

## Data Availability Statement

The datasets presented in this study can be found in online repositories. The names of the repository/repositories and accession number(s) can be found below: http://cgss.ruc.edu.cn/.

## Author Contributions

SG wrote and revised the manuscript and conducted the statistical analysis. JZ contributed to planning the study and revised the manuscript. Both authors contributed to the article and approved the submitted version.

## Conflict of Interest

The authors declare that the research was conducted in the absence of any commercial or financial relationships that could be construed as a potential conflict of interest.

## Publisher’s Note

All claims expressed in this article are solely those of the authors and do not necessarily represent those of their affiliated organizations, or those of the publisher, the editors and the reviewers. Any product that may be evaluated in this article, or claim that may be made by its manufacturer, is not guaranteed or endorsed by the publisher.

## Appendix

The results of confirmatory factor analysis for special social trust and general social trust: RMSEA = 0.074, CFI = 0.952, TLI = 0.925, SRMR = 0.039.
